# Control of Hepatitis A by Universal Vaccination of Adolescents, Puglia, Italy

**DOI:** 10.3201/eid1403.070900

**Published:** 2008-03

**Authors:** Pietro Luigi Lopalco, Rosa Prato, Maria Chironna, Cinzia Germinario, Michele Quarto

**Affiliations:** * University of Bari, Bari, Italy; † University of Foggia, Foggia, Italy

**Keywords:** Hepatitis A vaccine, mass vaccination, herd immunity, Italy, letter

## Abstract

Control of Hepatitis A by Universal Vaccination of Adolescents, Puglia, Italy

**To the Editor:** The incidence of hepatitis A in Italy has decreased in the past 2 decades because of improved sanitation and better living conditions (*1*). However, large outbreaks occurred in the 1990s in several southern regions of Italy, despite lower rates of infection among the general population (*2*–*4*). Person-to-person transmission has been recognized as a major factor in spread of this disease during this period (*5*).

Safe and highly effective hepatitis A vaccines have been available since 1995. Nevertheless, their use has been limited to the Western Hemisphere. Universal vaccination programs have been initiated only in the United States and Israel before 1998 (*6*). In 1998, after a large epidemic of hepatitis A, a vaccination program for toddlers and adolescents was initiated in Puglia in southeastern Italy, which has a population >4 million. This vaccine was offered free to all children 15–18 months of age and to adolescents 12 years of age. Until 2002, a combined hepatitis A plus B vaccine had been used for vaccination of adolescents as part of the national hepatitis B immunization program. In 2003, this hepatitis B vaccination program ended; only hepatitis A vaccines containing 1 antigen are now used. No catch-up vaccination campaign has been planned.

We analyzed disease surveillance and vaccine coverage data for 1991–2006 to evaluate the effect of such a vaccination program on hepatitis A incidence in persons in Puglia during the 9 years after initiation of the program. In the period before the vaccination program was initiated (1989–1997), annual incidence rates of hepatitis A in Puglia ranged from 4.3 to 139.8 cases/100,000. The average annual rate during this period was 49.5 cases/100,000. Two large outbreaks were reported in Puglia, the first in 1992 and the second in 1996–1997 (*5*). During the 9 years after start of the vaccination program (1998–2006), incidence of hepatitis A decreased from 22.8 cases/100,000 in 1998 to 0.7 cases/100,000 in 2006 (Figure). In the same period in other regions of Italy, incidence of hepatitis A was 5 cases/100,000, without any evident annual peak.

**Figure:**
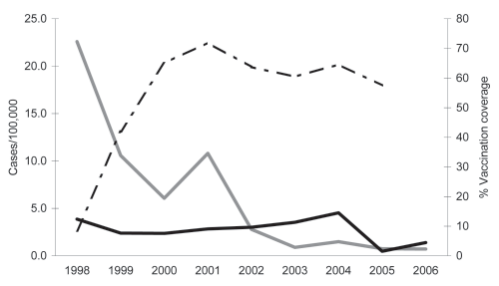
Incidence of hepatitis A in Puglia, Italy (gray line) compared with the rest of Italy (black line), 1998–2006, and hepatitis A vaccination coverage among adolescents in Puglia (dashed line), 1998–2005.

Since 2002, annual incidence rates in Puglia have remained at <2.8 cases/100,000, lower than those in the rest of Italy. This incidence has been observed in all age groups, without any differences between vaccinated and unvaccinated birth cohorts. Vaccination coverage among children 15–18 months of age was <20% during the period of the vaccination program. Coverage levels in adolescents reached 65% in the third year after the start of the program and then ranged from 57% to 72% (Figure).

Hepatitis A has been a serious public health problem in Puglia. This disease has had a detrimental effect on the local economy, which is based on tourism and trade of food products. However, since the vaccination program was started in 1998, disease incidence has decreased. During the study period, no other alternative prevention measures that could have had an effect on disease control were implemented.

High levels of vaccination coverage have not been achieved since the start of the campaign, and no catch-up vaccination program has been implemented. The decrease in hepatitis A incidence we observed involved all age groups, including those not covered by the vaccination program. This finding may indicate strong herd immunity, which would confirm what has been observed in other countries (*7*–*9*). However, there is uncertainty in interpreting current epidemiologic data. On the basis of available data, we cannot assess whether the current low incidence of hepatitis A in Puglia is caused by vaccination alone or in combination with other factors. We also cannot exclude the possibility that what we observed may have been an interepidemic period and that new episodes may occur in the future.

Our results indicate that local health authorities should be aware of possible increases in the incidence of hepatitis A in Puglia. An urgent catch-up vaccination program may be necessary to prevent future outbreaks. Moreover, a seroepidemiologic survey would be useful for assessing the size of the susceptible population and most vulnerable age groups.
